# Behavioral Profiles of Adolescent Alcohol-Preferring/Non-preferring (P/NP) and High/Low Alcohol-Drinking (HAD/LAD) Rats Are Dependent on Line but Not Sex

**DOI:** 10.3389/fnins.2021.811401

**Published:** 2022-01-13

**Authors:** Stina Lundberg, Erika Roman, Richard L. Bell

**Affiliations:** ^1^Department of Pharmaceutical Biosciences, Uppsala University, Uppsala, Sweden; ^2^Department of Anatomy, Physiology and Biochemistry, Swedish University of Agricultural Sciences, Uppsala, Sweden; ^3^Department of Psychiatry, Stark Neuroscience Research Institute, Indiana University School of Medicine, Indianapolis, IN, United States

**Keywords:** adolescence, alcohol non-preferring rats, alcohol-preferring rats, high alcohol-drinking rats, low alcohol-drinking rats, multivariate concentric square field (MCSF)

## Abstract

Initial contact with alcohol generally occurs during adolescence, and high consumption during this period is associated with increased risk for later alcohol (AUDs) and/or substance use disorders (SUDs). Rodents selectively bred for high or low alcohol consumption are used to identify behavioral characteristics associated with a propensity for high or low voluntary alcohol intake. The multivariate concentric square field™ (MCSF) is a behavioral test developed to study rodents in a semi-naturalistic setting. Testing in the MCSF creates a comprehensive behavioral profile in a single trial. The current aim was to examine the behavioral profiles of adolescent, bidirectionally selectively bred male and female high alcohol-consuming (P and HAD1/2) and low alcohol-consuming (NP and LAD1/2) rat lines, and outbred Wistar rats. Alcohol-naïve rats were tested once in the MCSF at an age between postnatal days 30 and 35. No common behavioral profile was found for either high or low alcohol-consuming rat lines, and the effect of sex was small. The P/NP and HAD2/LAD2 lines showed within pair-dependent differences, while the HAD1/LAD1 lines were highly similar. The P rats displayed high activity and risk-associated behaviors, whereas HAD2 rats displayed low activity, high shelter-seeking behavior, and open area avoidance. The results from P rats parallel clinical findings that denser family history and risk-taking behavior are strong predictors of future AUDs, often with early onset. Contrarily, the HAD2 behavioral profile was similar to individuals experiencing negative emotionality, which also is associated with a vulnerability to develop, often with a later onset, AUDs and/or SUDs.

## Introduction

Addiction is a complex disorder that is influenced by environmental factors and their interaction with a genetic make-up. Additionally, a vulnerability for addiction is associated with certain personality traits, such as impulsivity and sensation seeking, and share comorbidity with multiple psychiatric disorders, including anxiety and depression (Babor, 1992; Cloninger et al., 1996; Lesch and Walter, 1996; Merikangas et al., 1998; Kranzler and Rosenthal, 2003; Moss et al., 2007). For alcohol use disorders (AUDs), the genetic risk is around 50% (Verhulst et al., 2015; Reilly et al., 2017), reflecting the importance of a family history of AUDs as a risk factor (Reilly et al., 2017). To examine the heritability of a vulnerability to develop AUDs, bidirectional selective breeding strategies in laboratory animals have been used (Bell et al., 2012, 2016, 2017).

Breeding programs with bidirectional selection for high vs. low alcohol consumption have generated seven pairs of high/low alcohol-consuming rat-lines across the world: the University of Chile bibulous/abstainer (UChB/UChA) rats (Quintanilla et al., 2006), the Finnish ALKO alcohol/non-alcohol (AA/ANA) rats (Sommer et al., 2006), the Sardinian alcohol-preferring/non-preferring (sP/sNP) rats (Colombo et al., 2006), the Warsaw high-/low-preferring (WHP/WLP) rats (Dyr and Kostowski, 2008), the Indiana alcohol-preferring/non-preferring (P/NP), and high/low alcohol-drinking (HAD/LAD, replicates 1 and 2) rat lines (McBride et al., 2014). Selective breeding of P/NP and the HAD/LAD replicate rat lines was based on identical selection criteria. P/NP rats were selected from Wistar rats (Murphy et al., 2002) and the HAD/LAD replicate lines from N/NIH rats (Murphy et al., 2002). Selectively bred lines have made it possible to determine behavioral (Roman et al., 2012), neurobiological (Bell et al., 2012), and genetic (McBride et al., 2012; Bell et al., 2017) characteristics associated with selection for high vs. low voluntary alcohol consumption. The high alcohol-consuming lines are also useful for evaluating pharmaceutical candidates to treat AUDs (Bell et al., 2012, 2016, 2017). The existing literature indicates that studies of male and adult animals have predominated in the past, with a modest increase in studies of sex-dependent behavior more recently (Bell et al., 2014). In addition, the consequences of peri-adolescent alcohol drinking have been a more recent focus of study, and both P and HAD rats show increased peri-adolescent alcohol consumption in both continuous and binge-like paradigms (Bell et al., 2014). Nonetheless, little is known about alcohol-naïve adolescent behavior of these lines, especially in females, and such studies would provide further information regarding modeling of family history for AUDs during development (Bell et al., 2013, 2014).

The multivariate concentric square field™ (MCSF) test is a behavioral test based on an ethoexperimental foundation, and it is designed to generate a behavioral profile that assesses exploration, risk-associated, and shelter-seeking behaviors (Meyerson et al., 2006). This allows for detection of individual and treatment-associated differences during both adolescence and adulthood (e.g., Palm et al., 2014; Karlsson and Roman, 2016; Wille-Bille et al., 2018; Lundberg et al., 2019; Gore-Langton et al., 2021). The MCSF has previously been used to characterize differences between selectively bred line-pairs and within each selection criteria (P/NP, HAD/LAD replicates, sP/sNP, and AA/ANA) in adult males (Roman et al., 2007, 2012; Roman and Colombo, 2009). Within-pair differences varied among the pairs (Roman et al., 2007, 2012; Roman and Colombo, 2009), and substantial differences were found among the high alcohol-consuming lines (Roman et al., 2012). More recently, an adapted version of the MCSF was used to profile differences after short-term selective breeding based on alcohol consumption in adolescence. Here, the high alcohol-consuming line showed increased shelter-seeking and decreased risk-taking behaviors when tested in adolescence (Fernández et al., 2020). The same behavioral trends were seen in an earlier replicate of the same breeding procedure using the light-dark box (Fernández et al., 2017). In the present study, we aimed to use the MCSF to further examine the link between a genetic propensity for high or low alcohol consumption and the adolescent behavioral profile before first contact with alcohol by examining naïve male and female P/NP and HAD/LAD (replicates 1 and 2) rats. Additionally, a Wistar cohort was included to provide an outbred comparison to the selectively bred lines. The goal was to evaluate both differences based on selected alcohol consumption (within-pair differences) and differences within the same selection criteria, i.e., among all three high or low alcohol-consuming lines. The hypotheses were to find similar within-pair differences in the behavioral profiles as seen in the study of the adult animals (Roman et al., 2012) and that the HAD/LAD replicates would be similar within each selection criteria while differing from the P/NP lines.

## Materials and Methods

### Animals, Housing, and Ethics Statement

Subjects were high alcohol-consuming P, HAD1, and HAD2 rats; low alcohol-consuming NP, LAD1, and LAD2 rats delivered to the laboratory at the day of weaning (Indiana University School of Medicine, Indianapolis, IN, United States); and outbred Wistar rats (RccHan:WI, Envigo, Indianapolis, IN, United States), for details see Table 1. Animals were pair-housed, by sex and line, in opaque plastic cages (22 × 44 × 20 cm) with sawdust bedding and *ad libitum* access to food (7001 Teklad 4%, Envigo, Madison, WI, United States) and water in a temperature-controlled (24 ± 1°C) and humidity-controlled (∼50%) animal room on a reversed 12 h/12 h dark-light cycle, with dark onset at 10:00. The animals were identified by tail markings with a marker pen, which were darkened as needed. Animals were acclimated to their home-cages for 5 days before any procedures were initiated. Experimental procedures were conducted during the dark period of the dark-light cycle. The animal experimental protocol was approved by the Institutional Animal Care and Use Committee of the Indiana University School of Medicine and is consistent with NIH’s Guide for the Care and Use of Laboratory Animals (National Research Council, 2011).

**TABLE 1:** Details about the animals in the study; number of animals per sex, to which generation they belong, from how many litters they were derived, and how many animals from each litter entered the study.

Line	n/sex	Generation	Number of litters	Pups/litter and sex	Age at weaning or delivery (PND)
P	20	S82–S83	11	1–3	22–25
HAD1	19–20	S70	8	2–3	23–25
HAD2	20	S69	9	1–4	22–25
NP	12	S81–S82	6	2	23–27
LAD1	12	S70	6	1–4	24–26
LAD2	12	S69	11	2	22–25
Wistar	20	n.a.	n.a.	n.a.	22

*n.a., not applicable; PND, postnatal day.*

### The Multivariate Concentric Square Field™ Test

**FIGURE 1:**
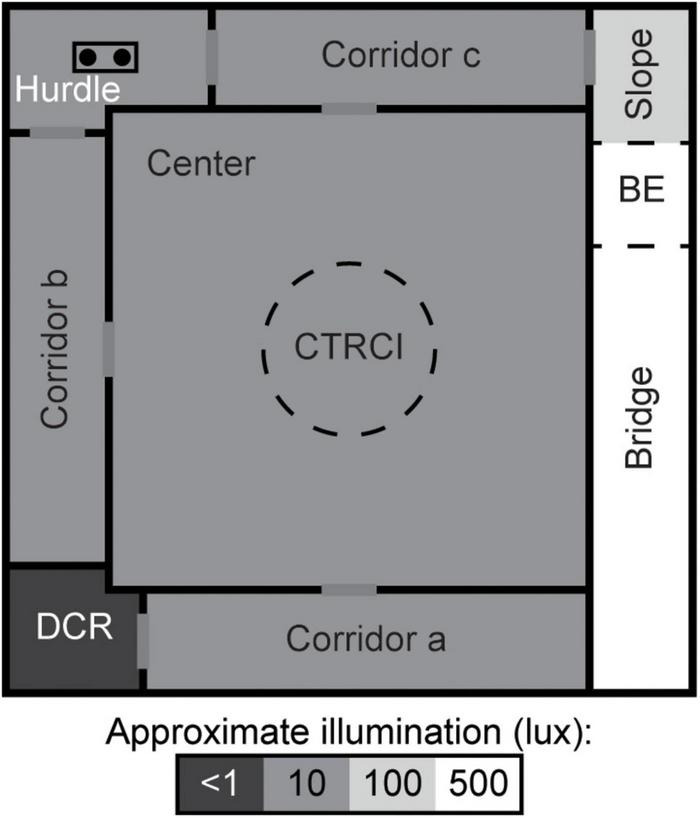
Schematic layout of the MCSF. The arena is divided into zones by walls (solid, black lines) or imagined boundaries (dashed lines). BE, bridge entrance; CTRCI, central circle; DCR, dark corner room. Modified with permission from Lundberg et al. (2019).

The MCSF (Figure 1) is described in detail elsewhere (Meyerson et al., 2006; Roman and Colombo, 2009). Briefly, the arena is 100 × 100 cm and divided into zones: the *center* (70 × 70 cm) with a *central circle* (CTRCI, 25 cm in diameter) surrounded on three sides by *corridors*. Along the fourth side, an elevated and illuminated “bridge” is divided into the *slope*, *bridge entrance* (BE), and *bridge*. One corner has a sheltered *dark corner room* (DCR) and another a raised *hurdle* with hole-board flooring. Light conditions in the different zones were as follows (lux): 15 in the CTRCI, 5–10 in the corridors as well as the hurdle, and 500 in the middle of the bridge.

Prior to testing, the animals were habituated to the handling procedures for three consecutive days. This consisted of individual handling, weighing, and adaptation to a small transportation cage. Between postnatal day (PND) 30 and 35, the animals were tested once in the MCSF. On the testing days, the animals to be tested were transported in their home cages, in conjunction with lights off, to a holding room. There, the animals were left undisturbed for 2 h before testing was initiated. The transportation cage was then used to transport the animals, one-at-a-time, to the testing room, and back after the test was concluded. First on each testing day, a non-test rat was allowed to explore the MCSF arena to avoid any first-to-be-tested effect. Males were always tested before females, and the two animals in a cage were never tested directly after each other. A trial started as the rat was released into the center of the arena facing the wall not leading to a corridor, and each trial lasted for 20 min. Between rats, the arena was sprayed with a 10% ethanol solution, wiped down, and allowed to air dry. After testing, all animals were returned to the animal room where they were weighed.

The trials were recorded by an overhead video camera and manually scored using EthoVision XT 13 (Noldus, Inc., Wageningen, Netherlands). The latency to first visit (L, s), frequency of visits (F, #), and duration spent (D, s) in each zone were scored together with the number of rearings, groomings, and stretched attend postures (SAPs). In addition, the number of urinations and fecal boli in the arena was counted after each trial. The frequency and duration spent in the corridors were summed into total corridor (totcorr) measures, and the latency (L, s) to leave the center after the start of the trial was identified. Further, the duration per visit (D/F, s), percental frequency (%F), and percental duration (%D) were calculated for each zone, including the total corridor measures. The total activity (TOTACT, i.e., sum of all frequencies), number of zones visited, and latency (L, s) to fully explore the arena and occurrences (Occ) for each zone not visited or behaviors not performed by all individuals in a group were also determined. For the corridors, only the total corridor measures were used for analysis, except for occurrences which were analyzed on the individual corridor level. Furthermore, the activity measures total activity and rearing were split into four 5-min periods for analysis of the activity profile over time during the trial.

### Statistical Analysis

Statistical analyses were carried out using Statistica 13 (TIBCO Software Inc., Tulsa, OK, United States) unless otherwise specified. Normality was examined with the Shapiro-Wilk’s test. Body weights were normally distributed, and differences were examined using a factorial ANOVA, with line and sex as between-subject factors, followed by *post hoc* Tukey HSD tests. The behavioral parameters from the MCSF were skewed and analyzed using non-parametric statistics. Continuous parameters were examined with Kruskal-Wallis ANOVA by ranks with *post hoc* Mann-Whitney *U*-tests with continuity corrections. Zone and behavior occurrences were analyzed with Maximum-Likelihood Chi-square test. Activity over time in the MCSF was analyzed in R 3.6.1 (R Core Team, 2019) with the nparLD package (Noguchi et al., 2012) with line and sex as between-subject factors and time as the within-subject factor. Time-dependent *post hoc* tests were performed with Wilcoxon’s matched pairs test and group-dependent tests with Mann-Whitney *U*-test with continuity corrections. The MCSF parameters were analyzed both with sex separated and with sex collapsed. All non-parametric *post hoc* comparisons evaluated: (1) differences within each line-pair, (2) differences within each selection criteria, and (3) differences between the Wistar rats and each of the selectively bred lines. Bonferroni corrected multiple comparisons were applied, and only one level of significance is reported (corresponding to *p* < 0.05). Effect sizes were calculated as previously described (Fritz et al., 2012).

Principal component analysis (PCA) and partial least squares projections to latent structures discriminant analysis (PLS-DA) were carried out in SIMCA 15 (Sartorius Stedim Data Analytics AB, Umeå, Sweden) as a multivariate complement to the standard statistics to further the multivariate interpretation of the MCSF. The PCA examined the relationship among one set of multiple variables, in this case MCSF parameters, and the PLS-DA examined the relationship between two sets of variables, one with multiple variables and one with a single categorical variable, in this case line. PCAs were carried out separately by line-pair to provide an overview of the relationship between each high and low alcohol-consuming line-pair, and PLS-DAs were carried out to examine the differences between all the lines. Autofit was used to generate the models; components were excluded if the eigenvalue was < 2.0 or if *Q*^2^ had a large negative value. Latencies, occurrences, percentage duration, and percentage frequency were not included in the models, and other parameters were excluded when advised by the software.

## Results

Five animals were excluded from the analyses: four due to technical issues during testing (two HAD1 females, one Wistar female, and one NP male) and one LAD1 female that spent > 70% of the trial climbing on the walls of the arena, which resulted in very little zone-dependent data.

Body weights for the animals at the time of testing are found in Supplementary Figure 1. Females weighed less than males in P, NP, and Wistar animals, while no sex-dependent differences were detected in HAD1, LAD1, HAD2, or LAD2 rats. Overall, the Wistar rats were heaviest, followed by P and then NP rats. While lower than P, NP, and Wistar rats, no body weight differences were detected across the HAD1, LAD1, HAD2, and LAD2 lines.

No sex-dependent differences were detected among the MCSF parameters in P, NP, HAD1, LAD1, or HAD2 animals. Among LAD2 animals, females urinated fewer times in the arena than males, and among Wistar animals, females had lower percentage frequency of visits to the center than males (Supplementary Table 1). Due to the weak effect of sex, further results are discussed with data collapsed across sex. However, for transparency, both numerical and statistical results are declared for each sex in Supplementary Table 1 together with the results with sex collapsed.

### Within Pair Comparisons

**FIGURE 2:**
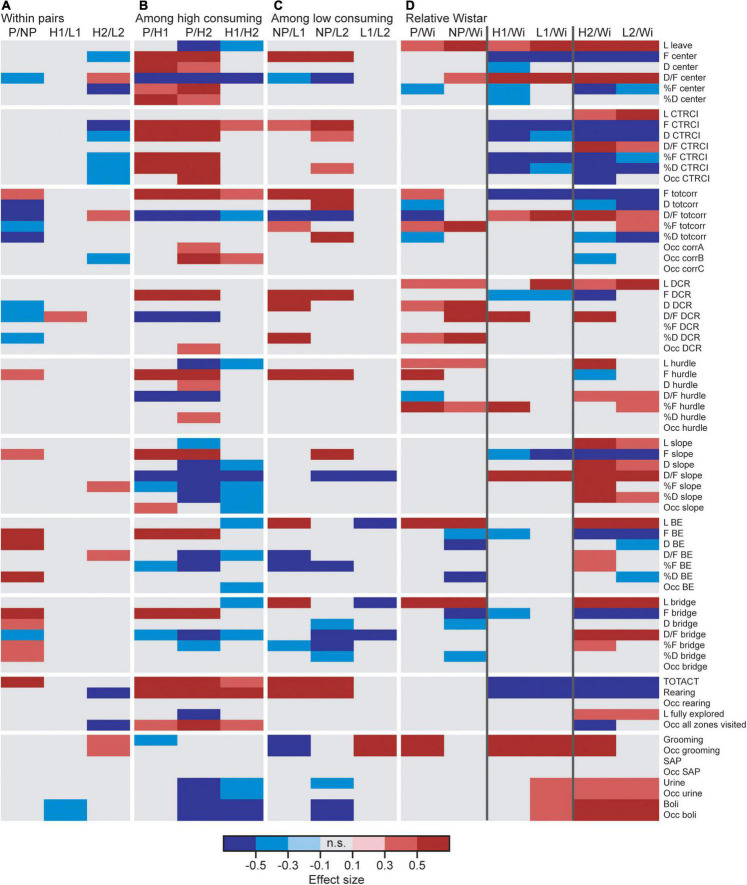
Heatmap of effect sizes for the significant differences comparing **(A)** the high and low alcohol-consuming rats within each selectively bred pair, **(B)** the three high consuming lines, **(C)** the three low consuming lines, and **(D)** the selectively bred lines relative to the outbred Wistar rats. P *n* = 40; NP *n* = 23; HAD1 *n* = 37; LAD1 *n* = 23; HAD2 *n* = 40, LAD2 *n* = 24, Wistar *n* = 39. BE, bridge entrance; CTRCI, central circle; corr, corridor; D, duration (s); DCR, dark corner room; D/F, duration per visit (s); F (#), frequency; H1/HAD1, high alcohol-drinking line, replicate 1; H2/HAD2, high alcohol-drinking line, replicate 2; L, latency (s); L1/LAD1, low alcohol-drinking line, replicate 1; L2/LAD2, low alcohol-drinking line, replicate 2; n.s., non-significant; NP, alcohol non-preferring line; Occ, occurrence (for zones not visited or behaviors not performed by all individuals); P, alcohol preferring line; SAP, stretched attend posture; TOTACT, total activity (i.e., sum of all frequencies); totcorr, total corridor; Wi, Wistar.

**FIGURE 3:**
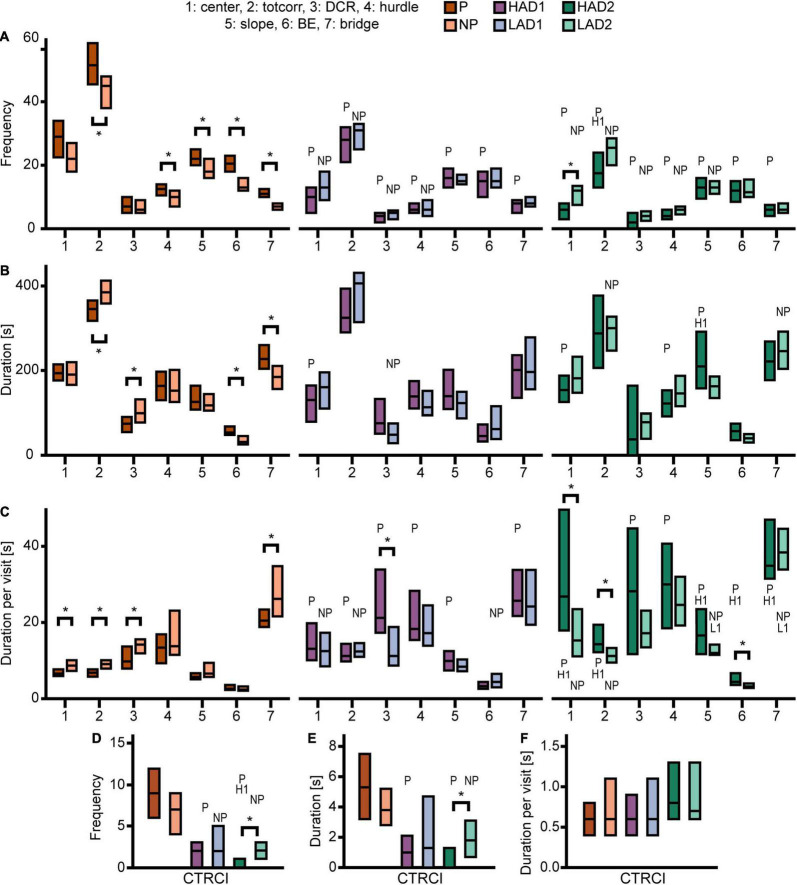
Results from the MCSF test in the selectively bred rats. Zone **(A)** frequency (#), **(B)** duration (s), and **(C)** duration per visit (s) in P, NP, HAD1, LAD1, HAD2, and LAD2 rats; **(D)** frequency (#), **(E)** duration (s), and **(F)** duration per visit (s) in the CTRCI for all lines. Data are presented as median with upper and lower quartiles. P *n* = 40; NP *n* = 23; HAD1 *n* = 37; LAD1 *n* = 23; HAD2 *n* = 40, LAD2 *n* = 24. *p < 0.0023 comparing high and low alcohol-consuming rats within each selectively bred pair; P *p* < 0.0023 relative to P rats; NP *p* < 0.0023 relative to NP rats; H1 *p* < 0.0023 relative to HAD1 rats (*post hoc* Mann-Whitney *U*-test with continuity correction). BE, bridge entrance; CTRCI, central circle; DCR, dark corner room; HAD1, high alcohol drinking line, replicate 1; HAD2, high alcohol-drinking line, replicate 2; LAD1, low alcohol-drinking line, replicate 1; LAD2, low alcohol-drinking line, replicate 2; NP, alcohol non-preferring line; P, alcohol preferring line; totcorr, total corridor.

**FIGURE 4:**
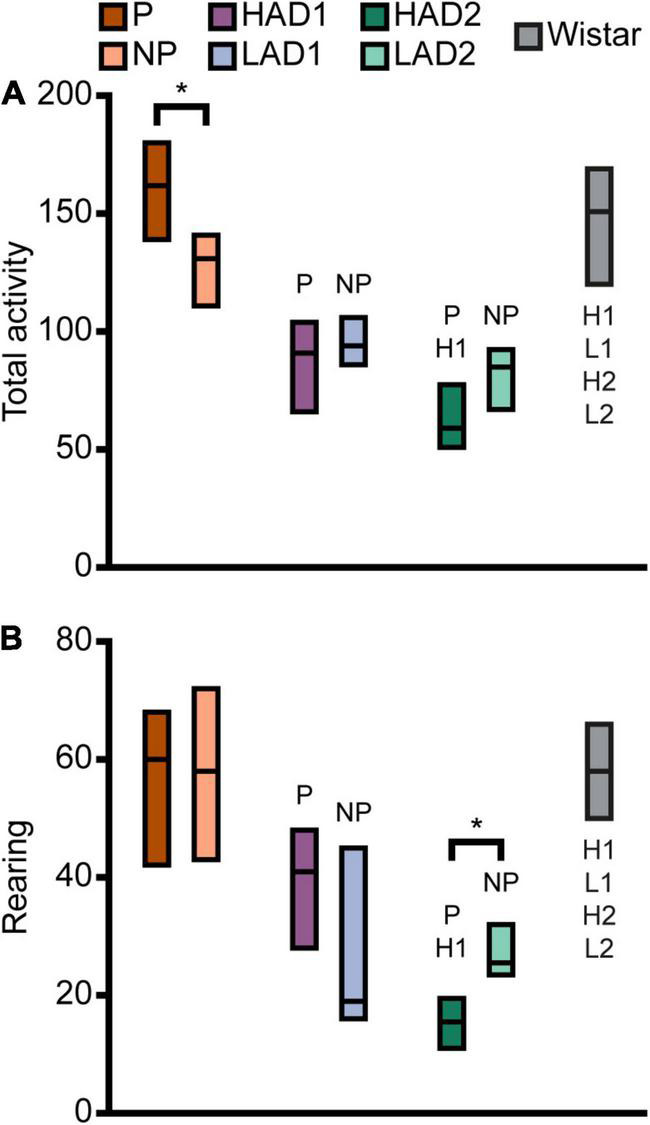
Activity in the MCSF expressed as **(A)** total activity (i.e., sum of all frequencies) and **(B)** rearing in the selectively bred lines and outbred Wistar rats. Data are presented as median with upper and lower quartiles. P *n* = 40; NP *n* = 23; HAD1 *n* = 37; LAD1 *n* = 23; HAD2 *n* = 40, LAD2 *n* = 24, Wistar *n* = 39. *p < 0.0023 comparing high and low alcohol-consuming rats within each selectively bred pair; P *p* < 0.0023 relative to P rats; NP *p* < 0.0023 relative to NP rats; H1 *p* < 0.0023 relative to HAD1 rats; L1 *p* < 0.0023 relative to LAD1 rats (*post hoc* Mann-Whitney *U*-test with continuity correction). HAD1, high alcohol-drinking line, replicate 1; HAD2, high alcohol-drinking line, replicate 2; LAD1, low alcohol-drinking line, replicate 1; LAD2, low alcohol-drinking line, replicate 2; NP, alcohol non-preferring line; P, alcohol preferring line.

Effect sizes for differences within each line-pair are presented in Figure 2A; results for frequencies, total duration, and duration per visit are displayed in Figure 3. The activity measures of total activity (i.e., sum of all zone frequencies) and rearing are shown in Figure 4 (totals for the 20-min trial) and Supplementary Figures 2A–F (over time in 5-min bins); and remaining results are found in Supplementary Table 1.

#### P and NP Rats

**FIGURE 5:**
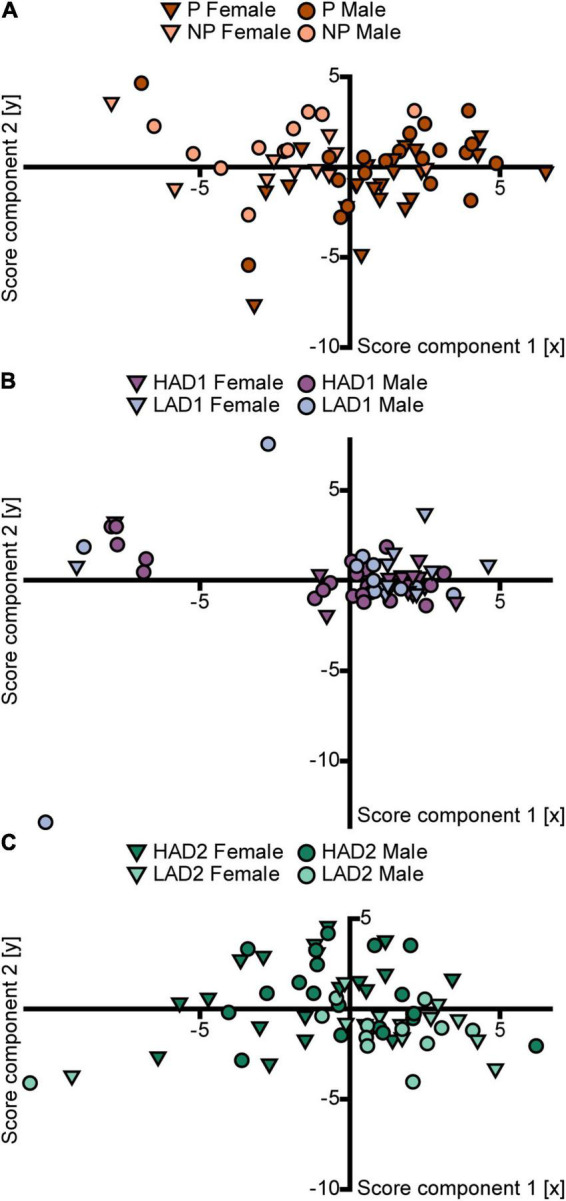
Scatter plots of the individual scores from the PCA analyses of the MCSF parameters in **(A)** P and NP rats [*n* = 63, 2 out of 4 components visualized, *R*^2^X_(1–2)_ = 0.46, *Q*^2^_(1–2)_ = 0.29], **(B)** HAD1 and LAD1 rats [*n* = 60, 2 out of 2 components visualized, *R*^2^X_(1–2)_ = 0.54, *Q*^2^_(1–2)_ = 0.27], and **(C)** HAD2 and LAD2 rats [*n* = 64, 2 out of 3 components visualized, *R*^2^X_(1–2)_ = 0.46, *Q*^2^_(1–2)_ = 0.24]. The plots are colored according to line and the shapes represent males (circles) and females (inverted triangles). The corresponding loading plots can be found in Supplementary Figure 3. HAD1, high alcohol-drinking line, replicate 1; HAD2, high alcohol-drinking line, replicate 2; LAD1, low alcohol-drinking line, replicate 1; LAD2, low alcohol-drinking line, replicate 2; NP, alcohol non-preferring line; P, alcohol preferring line.

The PCA analysis (*n* = 63, 4 components, *R*^2^X = 0.63, *Q*^2^ = 0.28) score plot (Figure 5A) confirmed the small effect of sex and indicated an influence of line on behavior in the MCSF by P and NP rats. Individual scores of P rats had higher positive loading on component 1 than the scores of NP rats, although some overlap between the lines was seen. Within each line, males and females displayed no separation. The parameter contributions to the analysis are displayed in the loading plot (Supplementary Figure 3A). The separation of P vs. NP scores occurred primarily in the first component (Figure 5A); P rat data were associated with total activity, zone frequencies (except the DCR), and duration at the bridge entrance and on the bridge. NP rat data were associated with duration spent in the corridors and duration per visit to the hurdle, slope, bridge entrance, and bridge (Supplementary Figure 3A).

Direct parameter comparisons showed that P rats visited the corridors, hurdle, slope, bridge entrance, and bridge more than NP rats (Figures 2A, 3A), which translated into higher total activity in P vs. NP rats (Figures 2A, 4A). P rats had shorter durations in the corridors and DCR, while having longer duration at the bridge entrance and bridge than NP rats (Figures 2A, 3B). The duration per visit was lower for P rats than NP rats in the center, corridors, DCR, and on the bridge (Figures 2A, 3C). Finally, P rats had lower percentage duration in the DCR, and lower percentage duration and frequency in the corridors than NP rats (Figure 2A and Supplementary Table 1). Instead, P rats had higher percentage duration at the bridge entrance, and higher percentage duration and frequency to the bridge than NP rats (Figure 2A and Supplementary Table 1).

When examining activity over time in the MCSF, as indicated by total activity and number of rearings (Supplementary Figures 2A,B, respectively), the pattern over time was similar between P and NP rats. P rats had higher total activity in the second and fourth 5-min periods than NP rats, while the total activity was comparable in the first and third 5-min periods. Both lines had lower initial activity that then sharply increased and peaked in the second 5-min period. For P rats, the total activity then declined to an intermediate level during the second half of the test. For NP rats, the decline was evident only in the fourth 5-min period, where it was comparable to the total activity during the initial 5 min (Supplementary Figure 2A). In number of rearing actions (Supplementary Figure 2B), no line-dependent differences were found. The number of rearings increased in P rats from the first to the second 5-min period; apart from this, no time-dependent differences were found within P or NP rats. In conclusion, P rats showed higher exploratory and risk-associated behaviors in the MCSF, while NP rats showed higher shelter-seeking behavior.

#### HAD1 and LAD1 Rats

The PCA analysis (*n* = 60, 2 components, *R*^2^X = 0.54, *Q*^2^ = 0.27) score plot (Figure 5B) shows a large overlap between the lines. Individual scores from both lines clustered together, indicating small variance within the main population, and an outlying group with a large negative contribution of component 1. Results in the loading plot (Supplementary Figure 3B) indicated that this outlying group had higher duration per visit to the DCR, hurdle, and slope. In addition, these outlying individuals did not visit all of the zones in the MCSF arena (Supplementary Data 1).

Direct parameter comparisons indicated a small difference within the line-pair. HAD1 and LAD1 rats did not differ in frequency nor duration in any of the zones (Figures 2A, 3A,B,D,E). Nor did the duration per visit differ between HAD1 and LAD1 rats (Figures 2A, 3C,F), except in the DCR where HAD1 rats had longer duration per visit than LAD1 rats (Figures 2A, 3C). The activity measures of total activity and rearing did not differ between HAD1 and LAD1 rats (Figures 4A,B). However, a lower proportion of HAD1 rats defecated in the arena, resulting in a lower fecal boli count in HAD1 vs. LAD1 rats (Figure 2A and Supplementary Table 1).

The HAD1 and LAD1 rats showed only minor changes in their activity (i.e., total activity and rearing) over time in the MCSF (Supplementary Figures 2C,D). LAD1 rats showed no time-dependent changes, while HAD1 rats had higher total activity in the second compared to the fourth 5-min period (Supplementary Figure 2C). HAD1 rats had a higher number of rearings in the second 5-min period than LAD1 rats (Supplementary Figure 2D). HAD1 rats also had a higher number of rearings in the second compared to the first and fourth 5-min periods (Supplementary Figure 2D). In conclusion, HAD1 and LAD1 rats had a high degree of similarity in their behavioral profiles as assessed with the MCSF.

#### HAD2 and LAD2 Rats

The PCA analysis (*n* = 64, 3 components, *R*^2^X = 0.56, *Q*^2^ = 0.20) score plot (Figure 5C) confirmed a small effect of sex and indicated an influence of line on behavior in the MCSF by HAD2 and LAD2 rats. Individual scores of HAD2 rats loaded widely across all four quadrants, while the scores of LAD2 rats clustered together predominantly in the lower right quadrant (Figure 5C), indicating lower variance in the LAD2 rat data. Within each line, males and females displayed no separation. The parameter contributions to the analysis are displayed in the loading plot (Supplementary Figure 3C). LAD2 rats were characterized by total activity, rearing, duration in the corridors, and frequency of visits to the center, CTRCI, corridors, DCR, and hurdle.

Direct parameter comparisons showed that HAD2 vs. LAD2 line differences were enriched among parameters relating to the center and CTRCI (Figure 2A and Supplementary Table 1). HAD2 rats had a lower frequency of visits to the center and CTRCI [in absolute numbers (Figures 3A,D) and by percentage frequency (Figure 2A and Supplementary Table 1)], a lower duration in the CTRCI [in absolute numbers (Figure 3E) and by percentage duration (Figure 2A and Supplementary Table 1)], and higher duration per visit to the center (Figures 2A, 3C) than LAD2 rats, and fewer HAD2 than LAD2 rats visited the CTRCI (Figure 2A and Supplementary Table 1). Furthermore, among the other parameters, HAD2 rats had higher durations per visit in the corridors and on the bridge entrance than LAD2 rats (Figures 2A, 3C). HAD2 rats also reared less (Figure 4B) and a lower proportion of HAD2 animals visited corridor “b” and all zones compared to LAD2 rats (Figure 2A and Supplementary Table 1). The percentage frequency of visits to the slope, the number of grooming bouts, and the proportion of animals grooming were higher in HAD2 than LAD2 rats (Figure 2A and Supplementary Table 1).

There were no differences between the lines in total activity in any of the 5-min periods (Supplementary Figure 2E). HAD2 rats increased in total activity from the first to the second and third 5-min periods, and the total activity during the second period was also higher than in the fourth 5-min period. LAD2 rats increased in total activity from the first to the second 5-min period, while no other time-dependent differences were seen. In rearing over time (Supplementary Figure 2F), HAD2 rats had lower number recorded during the first, second, and fourth 5-min periods than LAD2 rats. LAD2 rats were stable in their number of rearings over time, while HAD2 rats increased their number of rearings from the first to the second and third 5-min period. In conclusion, HAD2 rats had lower activity and higher avoidance of open areas than LAD2 rats in the MCSF test.

### Comparisons Among High Alcohol-Consuming Rat Lines

Effect sizes for differences among the high alcohol-consuming rat lines are presented in Figure 2B; results for frequencies, total duration, and duration per visit are displayed in Figure 3. The activity measures of total activity and rearing are displayed in Figure 4 (totals for the 20-min trial) and Supplementary Figures 2A–F (over time in 5-min periods); and remaining results are found in Supplementary Table 1.

P rats differed substantially from the two HAD lines. P rats had the highest number of visits to all zones (Figures 2B, 3A,D), which resulted in higher total activity than either HAD line (Figures 2B, 4A). P rats also reared more than either HAD line (Figures 2B, 4B). Moreover, P rats had a lower duration per visit to each zone than either HAD line (Figures 2B, 3C), except for the CTRCI, where the duration per visit did not differ among the lines (Figures 2B, 3F), and at the bridge entrance, the P rats had a lower duration per visit than HAD2, but not HAD1, rats (Figures 2B, 3C). P rats had higher duration in the center and CTRCI than either HAD line, whereas the duration was higher in the hurdle and lower in the slope in P vs. HAD2 rats (Figures 2B, 3B,E). A higher proportion of P rats visited the entire arena than either HAD line; a higher proportion of P rats visited the CTRCI, DCR, and two of the corridors than HAD2 rats; and the proportion of P rats visiting the slope was higher than that of HAD1 rats (Figure 2B and Supplementary Table 1). Lastly, P rats had a shorter latency in leaving the center at the start of the trial, shorter latency in fully exploring the entire arena, and shorter latencies in first entering the hurdle and slope than HAD2 rats, whereas no latencies differed between P and HAD1 rats on these measures (Figure 2B and Supplementary Table 1).

The two HAD replicates also showed some differences. The total activity was higher in HAD1 vs. HAD2 animals (Figures 2B, 4A), driven by higher frequency of visits to the corridors and CTRCI in HAD1 than HAD2 rats (Figures 2B, 3A,D). HAD1 rats also reared more than HAD2 rats (Figures 2B, 4B). HAD1 rats had shorter duration on the slope (Figures 2B, 3B) and shorter duration per visit in all zones except the DCR, hurdle, and CTRCI (Figures 2B, 3C,F) than HAD2 rats. The latency in leaving the center at the start of the trial and in first entry of the hurdle, bridge entrance, and bridge were lower in HAD1 than HAD2 rats (Figure 2B and Supplementary Table 1). For HAD1 rats, a higher proportion visited the entire arena and corridor “b,” while a lower proportion visited the slope and bridge entrance than the proportion of HAD2 rats (Figure 2B and Supplementary Table 1).

The total activity per 5-min period (Supplementary Figures 2A,C,E) was higher in P rats than either HAD line throughout the test, while the HAD1 and HAD2 lines were similar except in the initial 5 min where HAD1 rats had higher activity. In the pattern over time, P and HAD2 animals showed similar tendencies, although the actual frequency counts where approximately twice as high in P than HAD2 rats, with a clear increase to a peak total activity in the second 5-min period (Supplementary Figures 2A,E), as opposed to HAD1 rats that had a much flatter curve. The rearing over time (Supplementary Figures 2B,D,F) was also higher for P rats than either HAD line, except in the second period where P and HAD1 rats did not differ. HAD1 rats had higher frequency of rearing than HAD2 throughout the test. For rearing, the pattern over time was more similar among the lines, with increases between the first and second 5-min period in all three lines.

**FIGURE 6:**
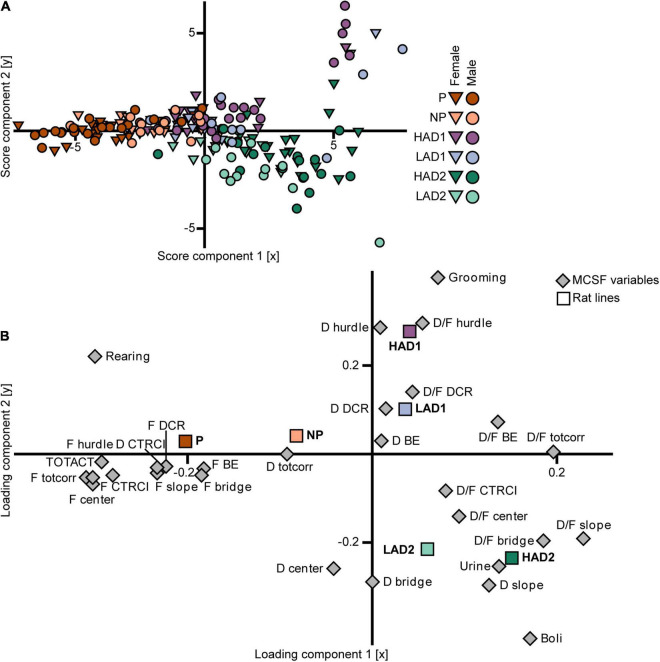
Scatter plots of **(A)** individual scores and **(B)** variable loadings from the PLS-DA [*n* = 187, 2 out of 3 components visualized, *R*^2^X_(1–2)_ = 0.47, *R*^2^Y_(1–2)_ = 0.21, *Q*^2^_(1–2)_ = 0.18] of the MCSF parameters (variable set 1) and selectively bred line (variable set 2). The plots are colored according to line and the shapes represent males (circles) and females (inverted triangles). BE, bridge entrance; CTRCI, central circle; D, duration; DCR, dark corner room; D/F, duration per visit; F, frequency; HAD1, high alcohol-drinking line, replicate 1; HAD2, high alcohol-drinking line, replicate 2; LAD1, low alcohol-drinking line, replicate 1; LAD2, low alcohol-drinking line, replicate 2; NP, alcohol non-preferring line; P, alcohol preferring line; TOTACT, total activity (i.e., sum of all zone frequencies); totcorr, total corridor.

In the PLS-DA analysis of the selectively bred lines (*n* = 187, 3 components, *R*^2^X = 0.57, *R*^2^Y = 0.26, *Q*^2^ = 0.22; Figure 6), the three high alcohol-consuming lines’ scores differed mainly in the first component (Figure 6A). The P rats had scores with a negative contribution on component 1, the scores for the HAD1 rats clustered around the origin, and the scores for the HAD2 rats had a largely positive contribution from component 1 as well as loading mostly in the lower right quadrant (Figure 6A). The HAD1 line had an outlying group in the upper right quadrant (Figure 6A), which constituted an outlying cluster in the PCA analysis as well (Figure 5B). In the loading plot (Figure 6B), the relationship between the class variable (line) in the form of aggregate dummy variables and the parameters (MCSF variables) is visualized. The P rat aggregate variable loaded with a large negative contribution from component 1 and a low contribution from component 2, which was associated with frequency of visits to all zones, total activity, duration in the CTRCI, and number of rearings. The HAD1 rat aggregate variable loaded in the upper right quadrant with a larger contribution from component 2 compared with component 1, which was associated with frequency of grooming, as well as duration and duration per visit to the hurdle and DCR. The HAD2 rat aggregate variable loaded in the lower right quadrant with relatively equal contributions from components 1 and 2, which was associated with duration on the slope, number of urinations and fecal boli, and duration per visit in the center, slope, and bridge (Figure 6B). In conclusion, there were no common behavioral profiles across the P and HAD replicate lines. P rats were highly active in the arena and showed increased risk-associated behavior, while HAD2 rats had low activity and increased risk-avoidance and shelter-seeking behavior. Lastly, the HAD1 rat data clustered between that of the other two high alcohol-consuming lines.

### Comparisons Among Low Alcohol-Consuming Rat Lines

Effect sizes for differences among the low alcohol-consuming rat lines are presented in Figure 2C; results for frequencies, total duration, and duration per visit are displayed in Figure 3. The activity measures of total activity and rearing are shown in Figure 4 (totals for the 20-min trial) and Supplementary Figures 2A–F (over time in 5-min periods); and remaining results are found in Supplementary Table 1.

The NP rats differed substantially from the two LAD lines. NP rats had a higher frequency of visits to the center, corridors, DCR, hurdle, and CTRCI zones than either LAD line and a higher frequency of visits to the slope than LAD2 rats (Figures 2C, 3A,D); this resulted in higher total activity in NP rats than either LAD line (Figures 2C, 4A). NP rats also reared more than either LAD line (Figures 2C, 4B). NP rats had a lower duration per visit to the center and corridors than either LAD line, as well as a lower duration per visit to the bridge entrance than the LAD1 rats and a lower duration per visit to the slope and on the bridge than LAD2 rats (Figures 2C, 3C). NP rats had a longer duration in the DCR than LAD1 rats, and a lower duration on the bridge and a higher duration in the corridors and CTRCI than LAD2 rats (Figures 2C, 3B,E). The NP rats had a higher latency to first entry of the bridge entrance and bridge than LAD1 rats (Figure 2C and Supplementary Table 1). Lastly, NP rats engaged in less grooming behavior than LAD1 rats and urinated fewer times, produced fewer fecal boli, and had a lower proportion of individuals defecating than LAD2 rats (Figure 2C and Supplementary Table 1).

The two LAD replicate lines were largely similar. The LAD1 and LAD2 rats did not differ in frequency or duration in any zone (Figures 2C, 3A,B) nor in total activity or rearing (Figures 2C, 4). However, LAD1 rats groomed more, had shorter latency to the bridge entrance and bridge (Figure 2C and Supplementary Table 1), and had lower duration per visit on the slope and bridge (Figures 2C, 3C) than LAD2 rats. When breaking down the total activity and rearing frequency in 5-min periods (Supplementary Figure 2), NP rats had higher scores than either LAD line at all time-points with a few exceptions; NP and LAD1 rats did not differ in total activity during the first 5-min period (Supplementary Figures 2A,C), and none of the low alcohol-consuming lines differed in rearing activity during the last 5-min period (Supplementary Figures 2B,F). In activity pattern overtime, the low alcohol-consuming lines were similar in rearing activity where all three lines had flat curves, which is in contrast to their high-consuming counterparts (Supplementary Figures 2B,D,F). Contrary, in total activity over time, there was more correspondence within each line-pair than among the low alcohol-consuming lines (Supplementary Figures 2A,C,D).

In the PLS-DA analysis (*n* = 187, 3 components, *R*^2^X = 0.57, *R*^2^Y = 0.26, *Q*^2^ = 0.22; Figure 6), the scores (Figure 6A) for the NP rats had a small to moderate negative contribution from component 1 and a low contribution from component 2. The scores for the LAD1 rats loaded around the origin with a few outlying individuals in the upper right quadrant, whereas the scores for the LAD2 rats loaded mostly in the lower right quadrant. The loading plot (Figure 6B) shows that the aggregate variables for the low alcohol-consuming lines loaded closer to the origin, while retaining a similar vector orientation, compared with their high alcohol-consuming counterparts, indicating that variance among the low alcohol-consuming lines was smaller than that of the high alcohol-consuming lines. The NP rat aggregate variable had a negative contribution from component 1 with minimal contribution from component 2. These results were associated with duration spent in the corridors and, to a lesser extent, the frequency of visits to each zone. The LAD1 rat aggregate variable loaded in the upper right quadrant, close to the origin, which was associated with duration per visit to the DCR, and duration in the DCR and bridge entrance. The LAD2 rat aggregate variable loaded in the lower right quadrant, which was associated with duration spent in the center and on the bridge, and duration per visit to the center and CTRCI. In conclusion, no common behavioral profiles were found across the NP and LAD replicate rat lines, although the differences were fewer than that observed between the high alcohol-consuming lines. The two LAD replicate rat lines showed a large degree of similarity, while NP rats had higher activity and exploration in the MCSF than either LAD rat line.

### Comparisons Relative to Wistar Rats

**FIGURE 7:**
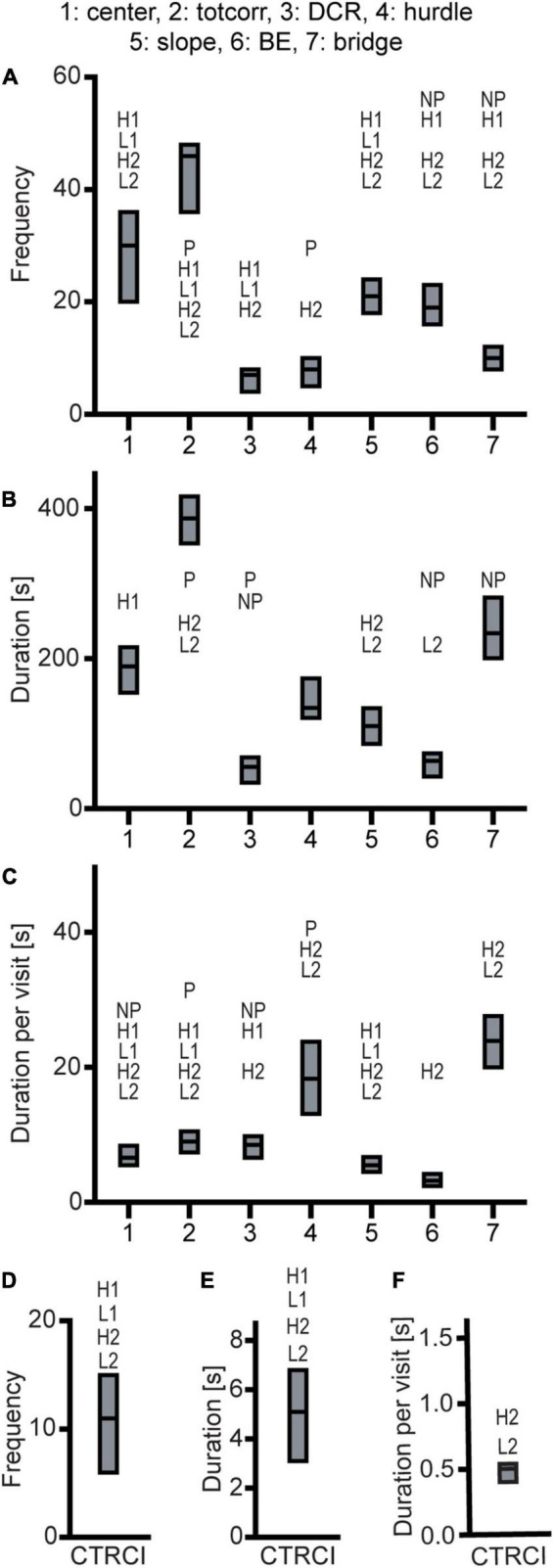
Results from the MCSF test in the outbred Wistar rats (*n* = 39). Zone **(A)** frequency (#), **(B)** duration (s), and **(C)** duration per visit (s); **(D)** frequency (#), **(E)** duration (s), and **(F)** duration per visit (s) in the CTRCI. Data are presented as median with upper and lower quartile. P *p* < 0.0023 relative to P rats; NP *p* < 0.0023 relative to NP rats; H1 *p* < 0.0023 relative to HAD1 rats; L1 *p* < 0.0023 relative to LAD1 rats; H2 *p* < 0.0023 relative to HAD2 rats; L2 *p* < 0.0023 relative to LAD2 rats (*post hoc* Mann-Whitney *U*-test with continuity correction). BE, bridge entrance; CTRCI, central circle; DCR, dark corner room; HAD1, high alcohol-drinking line, replicate 1; HAD2, high alcohol-drinking line, replicate 2; LAD1, low alcohol-drinking line, replicate 1; LAD2, low alcohol-drinking line, replicate 2; NP, alcohol non-preferring line; P, alcohol preferring line; totcorr, total corridor.

Effect sizes for differences relative to that of the outbred Wistar rats are presented in Figure 2D; results for frequencies, total duration, and duration per visit are displayed in Figure 7. The activity measures of total activity and rearing are shown in Figure 4 (totals for the 20-min trial) and Supplementary Figures 2G,H (over time in 5-min periods); and remaining results are found in Supplementary Table 1.

Wistar rats reared more during the trial than either HAD or LAD line, while they did not differ from the P or NP rats (Figures 2D, 4B). Wistar rats also had higher total activity than either HAD or LAD line, while they did not differ from the P or NP rats (Figures 2D, 4A). This is indicative of the differences seen in the zone frequencies (Figures 2D, 3A,D, 7A,D); Wistar rats had higher frequency than HAD1 rats in 7 out of 8 zones, than HAD2 in all zones, than LAD1 in 5 zones, and than LAD2 in 6 zones while only having higher frequency to the bridge entrance and bridge than NP rats and lower frequency to the corridors and hurdle than P rats. The same pattern is present for differences in duration per visit (Figures 2D, 3C,F, 7C,F); Wistar rats had lower duration per visit to most zones compared to the HAD and LAD lines, while the differences compared to P and NP rats were fewer. Differences among the zone durations were few overall (Figures 2D, 3B,E, 7B,E), but most noticeable were a higher duration in the CTRCI in Wistar vs. either HAD or LAD line, higher duration in the corridors in Wistar vs. P, HAD2 or LAD2 rats, lower duration in the DCR in Wistar vs. P or NP rats, and lower duration on the slope in Wistar vs. HAD2 or LAD2 rats. Overall, Wistar rats were the fastest to leave the center and start exploring the rest of the arena and had low latencies to many of the zones; however, the latency to fully explore the arena only differed between Wistar and HAD2 and LAD2 rats and Wistar rats differed in zone occurrences only relative HAD2 rats for two separate zones (Figure 2D and Supplementary Table 1).

When examining the total activity over time (Supplementary Figures 2A,C,E,G), Wistar rats had higher total activity than all selectively bred lines in the initial 5-min period and remained higher than either HAD or LAD line throughout the trial, except compared to LAD1 in the third 5-min period. From the second 5-min period, Wistar and NP rats had comparable total activity levels, while compared to P rats, Wistar rats had lower total activity level during the last two 5-min period. Over time, Wistar rats decreased their total activity from the first half of the trial to the second; this temporal pattern was unique to the Wistar rats. In rearing activity over time (Supplementary Figures 2B,D,F,H), Wistar rats had higher levels than either HAD or LAD line throughout the trial, except compared to HAD1 rats in the second 5-min period. Wistar rats did not differ in rearing activity over time compared to P or NP rats. Over time, rearing in Wistar rats increased from the first 5-min period compared to all subsequent periods. This pattern over time corresponded to a higher degree with the high alcohol-consuming lines, although no other line showed such a marked and sustained increase in rearing activity over the trial. In conclusion, the Wistar rats differed substantially from either HAD or LAD line, especially in overall exploration and risk-associated behaviors in the CTRCI. Differences compared to the P and NP lines were fewer but still pronounced, especially in activity at the start of the trial. The result of the PLS-DA analysis that included all of the rat lines (Supplementary Figure 4) supported this overall pattern; Wistar rats differed from all the selectively bred lines, although there were more similarities with the P and NP lines than with the HAD and LAD replicates.

## Discussion

In this study, the alcohol-naïve behavioral profiles of adolescent males and females from the Indiana selectively bred lines for high or low alcohol intake and preference (P/NP, HAD1/LAD1 and HAD2/LAD2) were examined using the MCSF test. The effect of sex on the behavioral profiles was minor across the rat lines, while there were considerable differences both within the line-pairs and between the selected phenotypes. The P/NP and HAD2/LAD2 line-pairs showed within-pair differences, while the HAD1 and LAD1 lines were highly similar. There was no common behavioral profile associated with either high or low alcohol-consuming phenotypes. The high alcohol-consuming lines differed substantially, especially in activity levels, where P rats showed the highest and HAD2 rats displayed the lowest activity. The low alcohol-consuming lines were more similar than their high alcohol-consuming counterparts, although the NP line differed from both LAD replicate lines, which were similar to each other.

### Behavioral Profiles of the P and NP Rat Lines

The present results revealed differences in adolescent behavior compared to previous studies with adult animals of the P and NP lines. An earlier study using the MCSF to profile adult male rats from five of the bidirectionally selectively bred line-pairs (including P/NP and HAD/LAD replicates) found lower activity in P than the NP rats (Roman et al., 2012). This contrasted with the present study where P rats had the highest levels of activity across all six lines. However, in studies using the open field test to assess locomotor activity, both adult male (Badishtov et al., 1995; Nowak et al., 2000; McKinzie et al., 2002; Roman et al., 2012) and female (McKinzie et al., 2002) P rats displayed higher locomotor activity than that seen in NP rats. Similar studies in adolescent or juvenile P/NP rats reported conflicting results; when tested at PND 33–40 P rats, independent of sex, had higher activity than NP animals after a saline injection (Rodd et al., 2004), whereas testing at PND 20 or 28 revealed no difference between P and NP rats (McKinzie et al., 2002). The present finding of higher risk-associated behavior in adolescent P rats also contrasts with earlier work that described adult P rats displaying behaviors interpreted as higher anxiety-like behavior (Stewart et al., 1993; Hwang et al., 2004; Pandey et al., 2005; Zhang et al., 2010), although other studies reported no such differences compared with adult NP rats (Badishtov et al., 1995; Viglinskaya et al., 1995; Roman et al., 2012). The previous study using the MCSF to profile adult selectively bred lines demonstrated different effects on risk-taking behavior in P vs. NP rats whether the difference in general activity was adjusted for or not. When the data was not adjusted for general activity, male P rats were less risk-taking than NP males. Contrarily, when the data was adjusted for general activity, male P rats had higher percentage frequency of visits to risk areas than NP males (Roman et al., 2012). In the present study, the results were in the same direction whether the analysis included an adjustment for general activity or not.

### Behavioral Profiles of the HAD and LAD Replicate Rat Lines

The replicate-dependent differences between the HAD/LAD lines also differed from previous studies. Using various behavioral tests, adult male HAD and LAD rats have been reported to be similar both within and between each replicate pair (Viglinskaya et al., 1995; Overstreet et al., 1997; Hwang et al., 2004). Similar observations have been reported for adolescent male and female rats (Rodd et al., 2004). However, differences have been reported between the HAD/LAD replicates using the open field and elevated plus maze tests (Roman et al., 2012). In the present study, the HAD2 rats differed from the other HAD/LAD lines with lower activity and higher risk avoidance, while the HAD1, LAD1, and LAD2 lines followed the main trend of similarity seen in the literature. Notably, HAD2 rats had lower risk-associated behavior in the CTRCI compared with the LAD2 rats. This has previously been observed among adult males of HAD1 vs. LAD1 rats (Roman et al., 2012). This previous MCSF study of the adult lines reported limited differences within each HAD/LAD line-pair, while showing more pronounced differences between the replicates: higher exploratory activity in HAD1 vs. HAD2 rats and lower risk-assessment behavior in LAD1 vs. LAD2 rats (Roman et al., 2012). Further interpretation of similarities to the adult behavior is difficult since previous work has shown that general and exploratory activity do not segregate well in the MCSF during adolescence and that risk-assessment behavior in the MCSF is expressed to a lower degree during adolescence than in adulthood (Lundberg et al., 2019). Support for a greater similarity between HAD1 vs. LAD1 rats than between HAD2 vs. LAD2 or P vs. NP rats was reported in a previous study examining the effects of repeated alcohol-deprivations on alcohol intake by LAD1, LAD2, and NP rats (Bell et al., 2004). This study revealed higher alcohol intake in LAD1 rats after multiple deprivations (Bell et al., 2004). This suggests that the separation in the selection phenotype between HAD1 vs. LAD1 rats may not be as stark as that observed between HAD2 vs. LAD2 or P vs. NP rats.

### Heterogeneity Within the Selection Criteria

That there are differences between the present adolescent results and previously reported adult behavior in the MCSF is not entirely surprising, as a previous study reported effects of age on behavior in the MCSF in outbred male Wistar rats (Lundberg et al., 2019). The present study indicates a similar pattern for P/NP behavior in the MCSF, which unexpectedly was the reverse of previous findings in adult P/NP males (Roman et al., 2012). However, the results from this previous study in male HAD/LAD rats (Roman et al., 2012) were more similar to the present results. Nowak et al. (2000) discussed apparent differences in the literature regarding interpretations of anxiety-like behavior (Stewart et al., 1993; Hwang et al., 2004; Pandey et al., 2005; Zhang et al., 2010) as well as locomotion and novelty-seeking behavior (Badishtov et al., 1995; Nowak et al., 2000; McKinzie et al., 2002; Roman et al., 2012) in P vs. NP rats, and to some extent HAD vs. LAD rats. They proposed that the findings may be connected to increased responsivity to novelty in P and HAD animals and a higher propensity to express “escape” rather than “exploratory” behaviors in a novel, inescapable environment (Nowak et al., 2000). However, this hypothesis is not supported by our findings, as P rats showed increased risk-associated behavior and increased activity, which may be attributed to higher novelty seeking and exploration. Also, the findings of low activity and high shelter-seeking behavior in HAD2 rats contradict this hypothesis (Nowak et al., 2000). Thus, further research is needed to tease apart these phenotypic subtypes.

The present results of differences between P and HAD lines may be explained by the fact that these rat lines were derived from different foundation stocks: the P/NP lines from Wistar rats and the HAD/LAD lines from N/NIH rats (Murphy et al., 2002; Bell et al., 2012, 2016). This can also be supported by the results herein comparing the selectively bred lines with Wistar rats where the P/NP rats were more similar to the Wistar rats than the HAD/LAD lines. However, there is evidence of considerable variation within Wistar rats which are dependent on supplier origin, e.g., in behavior and voluntary alcohol intake (Palm et al., 2011a,b, 2012; Goepfrich et al., 2013; Momeni et al., 2015; Wood et al., 2016). Considering there has been over 40 years of breeding since the establishment of the P/NP lines (Lumeng et al., 1977), it is not certain that the similarities between P/NP and Wistar rats herein are due to shared lineage. Unfortunately, the N/NIH rats are no longer commercially available and are only maintained in small research colonies, therefore we were unable to include them as additional comparisons in this study. Due to the different foundations stocks, different genes for different behavioral traits are likely to have co-segregated with the genes for alcohol intake and preference in the different line-pairs (e.g., Tabakoff et al., 2009; McBride et al., 2012, 2013; Saba et al., 2015). The present results of differences between HAD1 and HAD2 rats, as opposed to previous research, may be a recent development between the now separate breeding colonies for the line-pairs or represent a previously undetected difference in adolescent behavior that has been present throughout the history of these rat lines.

In a previous study characterizing alcohol-naïve, adult male behavior in the MCSF, the Finnish AA and Sardinian sP lines (not included in this study) constituted opposing behavioral extremes, while the P and HAD1/2 lines were intermediate and more similar (Roman et al., 2012). The adolescent behavior of the P and HAD1/2 lines seems to be more dissimilar than their adult counterparts. Interestingly, the lines from the short-term selective breeding based on alcohol consumption in adolescence also demonstrated a clear difference in the adolescent behavioral profile (Fernández et al., 2020), where the high alcohol-consuming line seems to be most similar to the HAD2 profile in the current study. This highlights the importance to study the alcohol-naïve adolescent behavior of several high alcohol-consuming lines to investigate the full spectrum of associated phenotypes, i.e., subtypes of AUDs modeled by these selected lines.

## Concluding Remarks

Importantly, individual rat line-pairs selectively bred for high or low voluntary alcohol intake and preference do not necessarily represent the full spectrum of AUDs. A corollary is that differences found within any individual line-pair does not necessarily represent all phenotypic differences observed between individuals who are family history positive (FHP) or negative (FHN) for AUDs. Instead, the selectively bred rat lines appear to represent individual and/or subgroup differences, primarily within individuals who are FHP for AUD, which support the clinical diagnosis of AUDs as a spectrum disorder rather than an either-or phenomenon (Crabbe et al., 2010; Froehlich, 2010). Overall, the current findings support the heterogeneity of genotypes and phenotypes associated with a risk for developing AUDs. This stems from our observation that there does not appear to be a common behavioral “pathway” linking these selectively bred rat lines, especially during adolescence. Nevertheless, the behavioral profiles of the high alcohol-consuming lines can, as outlined above, provide important information about different AUD subtypes. For instance, the behavioral profile of the P rat has some parallels with clinical findings observed in individuals with a denser family history of AUDs, including risk-taking behavior and an earlier onset of AUD behavior (Cloninger et al., 1996; Moss et al., 2007). In contrast, the HAD2 behavioral profile may be interpreted as resembling individuals with higher negative emotionality, which has been associated with a vulnerability to develop AUDs and/or SUDs with later onset (Cloninger et al., 1996; Merikangas et al., 1998; Moss et al., 2007). Thus, studies with different selectively bred line-pairs can be expected to aid in the process of tailoring treatments, including pharmacological, to different subtypes of AUDs and thereby increasing treatment success rates.

## Data Availability Statement

The original contributions presented in the study are included in the article/Supplementary Material, further inquiries can be directed to the corresponding author/s.

## Ethics Statement

The animal study was reviewed and approved by the Institutional Animal Care and Use Committee of the Indiana University School of Medicine, Indianapolis, United States.

## Author Contributions

ER, RB, and SL were responsible for the study concept and design, provided critical revision of the manuscript for important intellectual content, and contributed to interpretation of findings. SL contributed to the data collection, performed the data analysis, and drafted the manuscript. RB provided resources and funding. All authors critically reviewed content and approved the final version for publication.

## Conflict of Interest

The authors declare that the research was conducted in the absence of any commercial or financial relationships that could be construed as a potential conflict of interest.

## Publisher’s Note

All claims expressed in this article are solely those of the authors and do not necessarily represent those of their affiliated organizations, or those of the publisher, the editors and the reviewers. Any product that may be evaluated in this article, or claim that may be made by its manufacturer, is not guaranteed or endorsed by the publisher.
