# Supplementary material for: Behavioral Profiles of Adolescent Alcohol-Preferring/Non-preferring (P/NP) and High/Low Alcohol-Drinking (HAD/LAD) Rats Are Dependent on Line but Not Sex
Source: Front Neurosci. 2022 Jan 13;15:811401. doi: 10.3389/fnins.2021.811401 (PMC8793359; doi:10.3389/fnins.2021.811401)
Supplement: Supplementary file 3 [file Image_1.PDF]

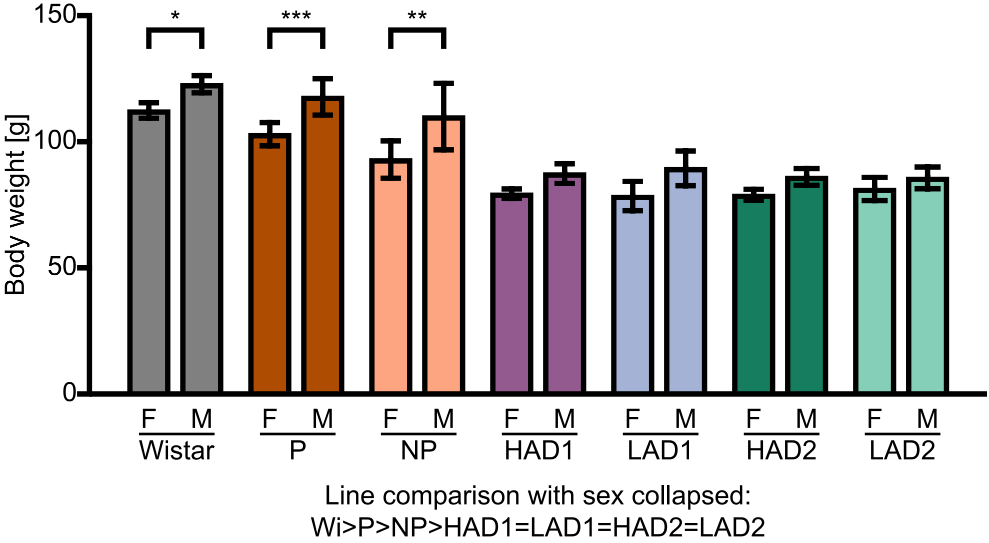

Figure S1. Body weights (g) of the animals at the time of testing in the MCSF. Data are presented as mean with 95% confidence interval.

P n=20/sex; NP n=11–12/sex; HAD1 n=17–20/sex; LAD1 n=11–12/sex; HAD2 n=20/sex, LAD2 n=12/sex, Wistar n=19–20/sex. *$p<0.05$, **$p<0.01$, ***$p<0.001$ (post hoc Tukey HSD test). F, female; HAD1, high alcohol-drinking line, replicate 1; HAD2, high alcohol-drinking line, replicate 2; LAD1, low alcohol-drinking line, replicate 1; LAD2, low alcohol-drinking line, replicate 2; M, male; NP, alcohol non-preferring line; P, alcohol preferring line; Wi, Wistar.
