# Supplementary material for: Behavioral Profiles of Adolescent Alcohol-Preferring/Non-preferring (P/NP) and High/Low Alcohol-Drinking (HAD/LAD) Rats Are Dependent on Line but Not Sex
Source: Front Neurosci. 2022 Jan 13;15:811401. doi: 10.3389/fnins.2021.811401 (PMC8793359; doi:10.3389/fnins.2021.811401)
Supplement: Supplementary file 4 [file Image_2.PDF]

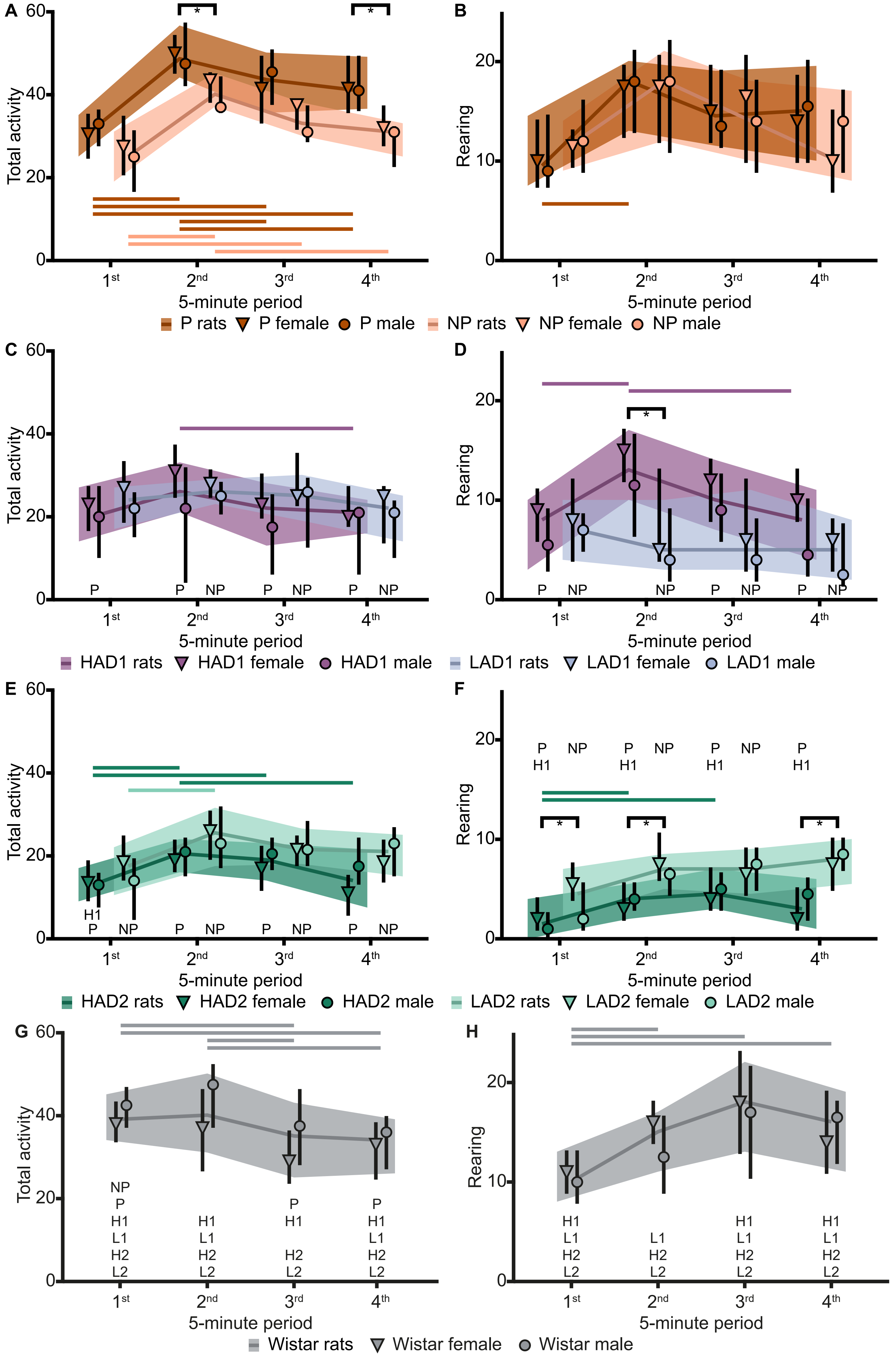

Figure S2. Activity over time in the MCSF expressed as total activity (i.e., sum of all frequencies) and rearing in 5-minute periods over the behavioral trial in A–B) P and NP rats, C–D) HAD1 and LAD1 rats, E–F) HAD2 and LAD2 rats, and G–H) Wistar rats. Data are presented as median with upper and lower quartiles for each line and by line×sex, statistics only declared for lines with sex collapsed. *$p<0.0018$ comparing high and low alcohol-consuming rats within each selectively bred pair; P $p<0.0018$ relative to P rats; NP $p<0.0018$ relative to NP rats; H1 $p<0.0018$ relative to HAD1 rats; L1 $p<0.0018$ relative to LAD1 rats; H2 $p<0.0018$ relative to HAD2 rats; L2 $p<0.0018$ relative to LAD2 rats (post hoc Mann-Whitney U-test with continuity correction). The colored lines denote time-dependent differences with $p<0.0018$ within each line (post hoc Wilcoxon's matched pairs test). HAD1, high alcohol-drinking line, replicate 1; HAD2, high alcohol-drinking line, replicate 2; LAD1, low alcohol-drinking line, replicate 1; LAD2, low alcohol-drinking line, replicate 2; NP, alcohol non-preferring line; P, alcohol preferring line
