# Supplementary material for: Behavioral Profiles of Adolescent Alcohol-Preferring/Non-preferring (P/NP) and High/Low Alcohol-Drinking (HAD/LAD) Rats Are Dependent on Line but Not Sex
Source: Front Neurosci. 2022 Jan 13;15:811401. doi: 10.3389/fnins.2021.811401 (PMC8793359; doi:10.3389/fnins.2021.811401)
Supplement: Supplementary file 5 [file Image_3.pdf]

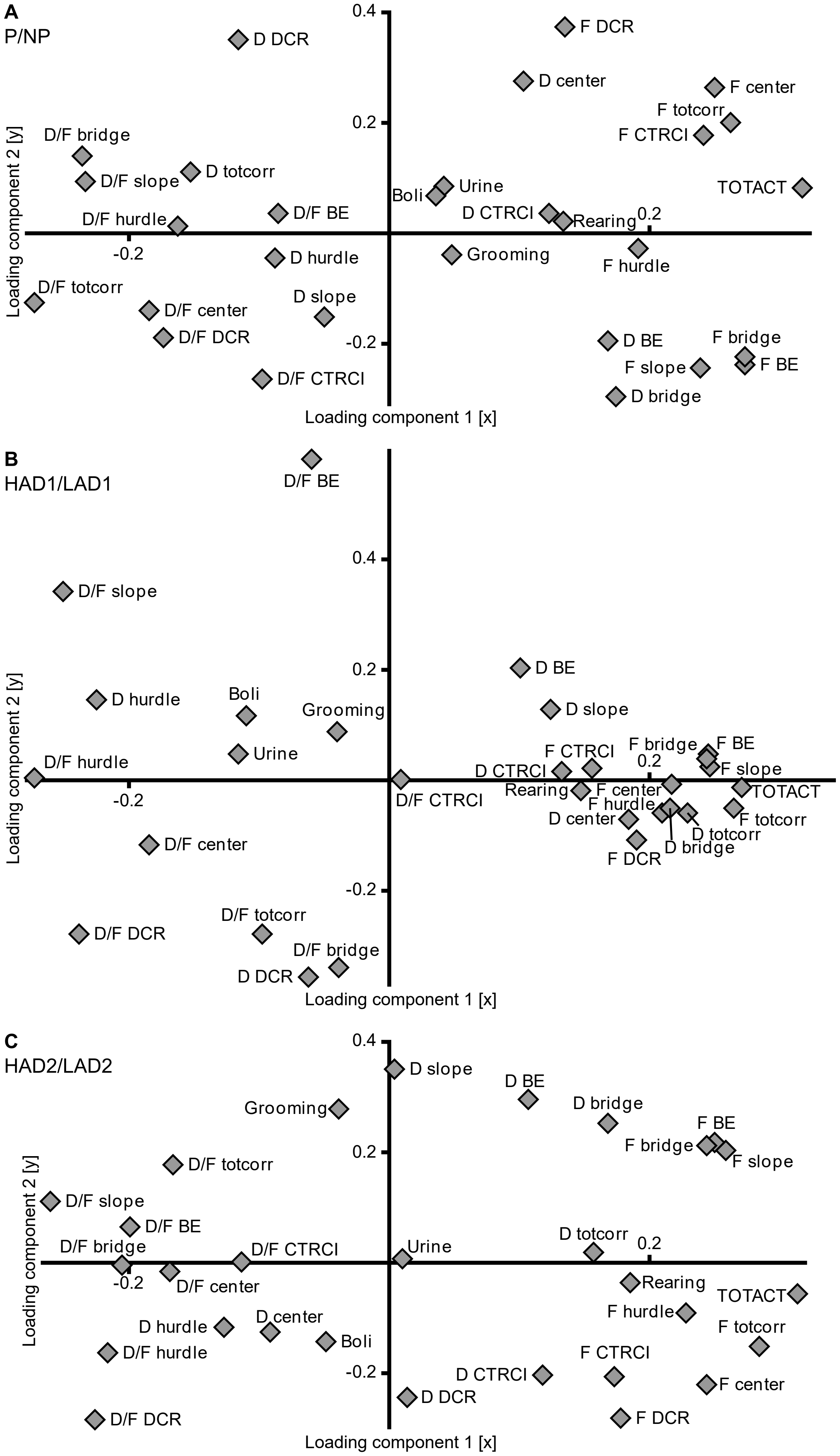

Figure S3. Scatter plots of the variable loadings from the PCA analyses of the MCSF parameters in A) P and NP rats (n=63, 2 out of 4 components visualized, $R^2X_{(1-2)}$=0.46, $Q^2_{(1-2)}$=0.29), B) HAD1 and LAD1 rats (n=60, 2 out of 2 components visualized, $R^2X_{(1-2)}$=0.54, $Q^2_{(1-2)}$=0.27) and C) HAD2 and LAD2 rats (n=64, 2 out of 3 components visualized, $R^2X_{(1-2)}$=0.46, $Q^2_{(1-2)}$=0.24). The corresponding score plots can be found in Fig. 5. BE, bridge entrance; CTRCI, central circle; D, duration; DCR, dark corner room; D/F, duration per visit; F, frequency; HAD1, high alcohol-drinking line, replicate 1; HAD2, high alcohol-drinking line, replicate 2; LAD1, low alcohol-drinking line, replicate 1; LAD2, low alcohol-drinking line, replicate 2; NP, alcohol non-preferring line; P, alcohol preferring line; TOTACT, total activity (i.e., sum of all zone frequencies); totcorr, total corridor.
