# Supplementary material for: Behavioral Profiles of Adolescent Alcohol-Preferring/Non-preferring (P/NP) and High/Low Alcohol-Drinking (HAD/LAD) Rats Are Dependent on Line but Not Sex
Source: Front Neurosci. 2022 Jan 13;15:811401. doi: 10.3389/fnins.2021.811401 (PMC8793359; doi:10.3389/fnins.2021.811401)
Supplement: Supplementary file 6 [file Image_4.PDF]

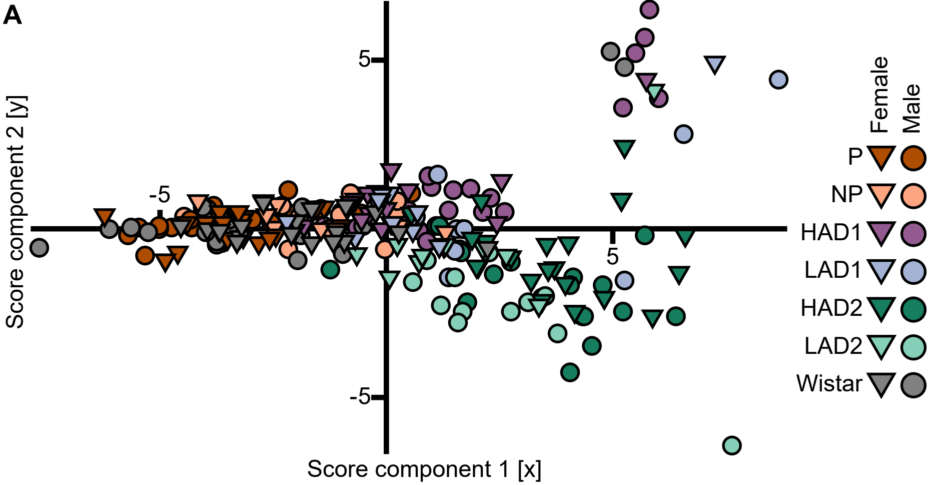

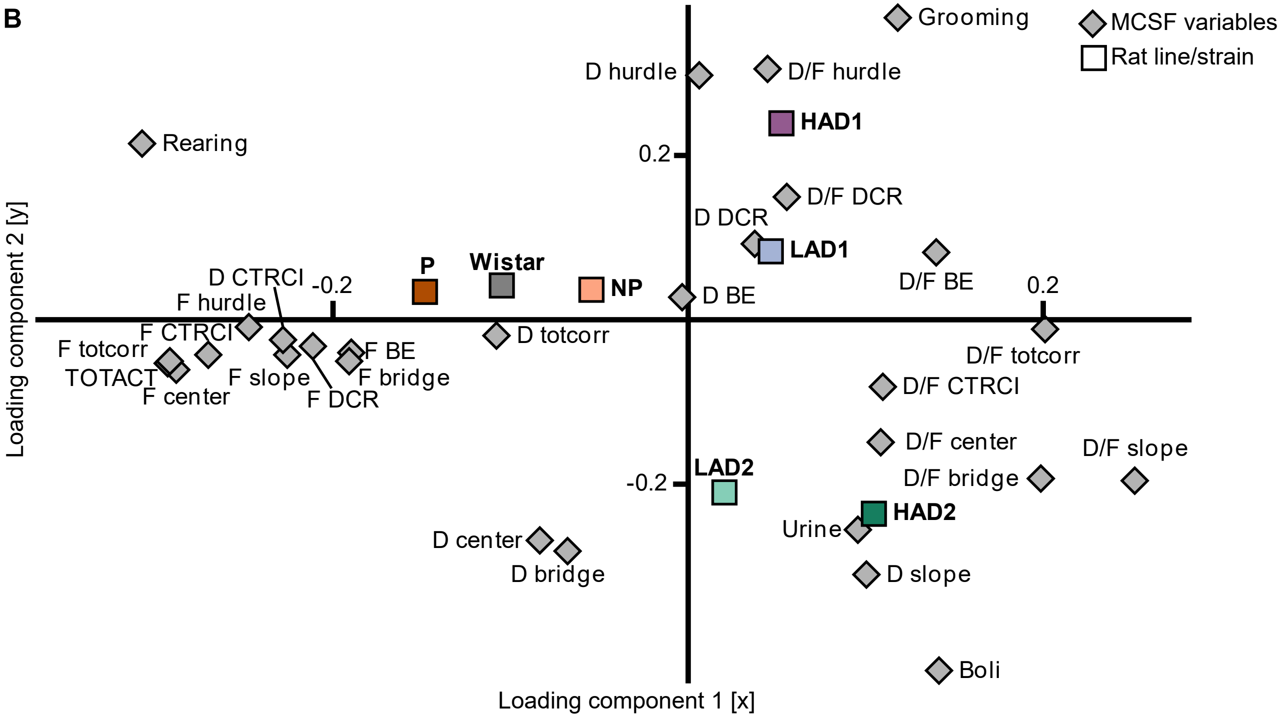

Figure S4. Scatter plots of A) individual scores and B) variable loadings from the PLS-DA (n=226, 2 out of 3 components visualized, $R^2X_{(1-2)}=0.48$, $R^2Y_{(1-2)}=0.16$, $Q^2_{(1-2)}=0.14$) of the MCSF parameters and selectively bred line or outbred strain. The plots are colored according to line/strain and the shapes represent males (circles) and females (inverted triangles). BE, bridge entrance; CTRCI, central circle; D, duration; DCR, dark corner room; D/F, duration per visit; F, frequency; HAD1, high alcohol-drinking line, replicate 1; HAD2, high alcohol-drinking line, replicate 2; LAD1, low alcohol-drinking line, replicate 1; LAD2, low alcohol-drinking line, replicate 2; NP, alcohol non-preferring line; P, alcohol preferring line; TOTACT, total activity (i.e., sum of all zone frequencies); totcorr, total corridor.
